# Second generation registry framework

**DOI:** 10.1186/1751-0473-9-14

**Published:** 2014-06-20

**Authors:** Matthew I Bellgard, Lee Render, Maciej Radochonski, Adam Hunter

**Affiliations:** 1 Centre for Comparative Genomics, Murdoch University, Murdoch, WA 6150, Australia

**Keywords:** Patient registry, Born digital, Data element, Genotype, Phenotype, Ontology

## Abstract

**Background:**

Information management systems are essential to capture data be it for public health and human disease, sustainable agriculture, or plant and animal biosecurity. In public health, the term patient registry is often used to describe information management systems that are used to record and track phenotypic data of patients. Appropriate design, implementation and deployment of patient registries enables rapid decision making and ongoing data mining ultimately leading to improved patient outcomes. A major bottleneck encountered is the static nature of these registries. That is, software developers are required to work with stakeholders to determine requirements, design the system, implement the required data fields and functionality for each patient registry. Additionally, software developer time is required for ongoing maintenance and customisation. It is desirable to deploy a sophisticated registry framework that can allow scientists and registry curators possessing standard computing skills to dynamically construct a complete patient registry from scratch and customise it for their specific needs with little or no need to engage a software developer at any stage.

**Results:**

This paper introduces our second generation open source registry framework which builds on our previous rare disease registry framework (RDRF). This second generation RDRF is a new approach as it empowers registry administrators to construct one or more patient registries without software developer effort. New data elements for a diverse range of phenotypic and genotypic measurements can be defined at any time. Defined data elements can then be utilised in any of the created registries. Fine grained, multi-level user and workgroup access can be applied to each data element to ensure appropriate access and data privacy. We introduce the concept of derived data elements to assist the data element standards communities on how they might be best categorised.

**Conclusions:**

We introduce the second generation RDRF that enables the user-driven dynamic creation of patient registries. We believe this second generation RDRF is a novel approach to patient registry design, implementation and deployment and a significant advance on existing registry systems.

## Background

The need for information systems, or registries in scientific disciplines is ubiquitous. For instance, in human rare disease [1-4] as well as plant and animal biosecurity [5,6]. In the field of human rare diseases alone, registries are used for clinical trial recruitment, surveillance, patient contact, natural disease history and longitudinal patient phenotyping. Previously, a first generation rare disease registry framework (RDRF), was developed to simplify the development and deployment of rare disease registries as there are over 6000 human rare conditions [3,4]. This framework introduced a modular design to rare disease registry development to reduce the amount of software developer time required to develop a new registry and enable the ability to incorporate new functionality efficiently. The RDRF introduced concepts including multi-level secure access, workgroups and simplifying the customisation of modules to enable reuse of component from one rare disease registry to the next across a range of registry requirements. The RDRF has been successfully deployed for a number of diseases both nationally and internationally [3,7].

However, within the RDRF there are a number of bottlenecks. For instance, for every new registry, data elements must be implemented at a programmatic level. That is, the registry schema and data elements are static. This leads to a number of limitations, for instance, i) while software developer code might be efficiently shared between registry implementations via modules, there is no simple, systematic way to link them at the user level, except through import/export functionality - a lower form of interoperability [4]; and ii) there is no ability to import (computer readable) standardised, predefined data elements (e.g. data elements defined within the Neurological Disorders and Stroke Common Data Element Project [8]). Software developer effort is required at every level of development and customisation. While the first generation RDRF is suitable for a number of use cases, it is recognised that community-wide ease of uptake without the need for software developer time is an important requirement that must be met.

It is not scalable for a software developer to be intimately involved in every single registry development and deployment. For instance, in a rare disease context, there are over 6000 rare conditions that need to be captured in multiple registries, in multiple countries, regions or jurisdictions. With the current state of registry development, there is no intuitive way to aggregate disease registries nor is it possible to easily reuse a data element (DE) created in one registry, into another. In a desirable scenario, for ten different national/international rare disease registries it should be possible to define a single Date of Birth (DOB) DE that can be reused in each of the ten registries. The advantages of this level of abstraction can be extended further. If a DE such as the DOB field is defined in a standardised template (e.g. [8]) with a unique identifier, then it would be feasible to dynamically import these standard DEs into a registry. In addition, in order to capture a new measurement, a new DE could be defined and dynamically added to a production registry well after the registry has been created.

In this paper, we describe this significant advance of the RDRF over static registry development in general. We outline the features of this second generation registry framework that enables non-software developers to dynamically create and administer patient registries.

## Methods

### System architecture

The registry is a web-based client server application utilising the following technologies: Django (https://www.djangoproject.com/), a Python based web framework; PostgreSQL (http://www.postgresql.org/), a relational database used to store framework metadata fields, user information, registry membership information and access permissions; MongoDB (http://www.mongodb.org/), a NoSQL schema-less document store, used to store registry-specific data for each patient; HTML, CSS, YAML (http://www.yaml.org/) and Javascript. The open source libraries jQuery (http://jquery.com/) and Bootstrap (http://getbootstrap.com/) are also used.

### Registry deployment

Currently RDRF is deployed to the CentOS (http://www.centos.org/) Linux operating system, using Apache (http://httpd.apache.org/) as a web server. Deploying using other operating systems and Linux distributions is possible, however currently only CentOS is tested and supported. A YUM (http://yum.baseurl.org/) repository and RPM (http://rpm.org/) for installation of the framework are also provided for CentOS 6 (http://rare-disease-registry-framework.readthedocs.org/).

## Results

We describe a highly dynamic web framework for the creation of patient registries with no extra software development. That is, users of the system do not need to be software developers to construct and deploy registries from scratch. To enable this, we exploited the metaprogramming features exposed by Python and the schema-less storage facilities provided by MongoDB.

### Meta-programming

Python allows creation of classes as first class citizens. By constructing classes at runtime, the RDRF is able to construct complex dynamic forms based on user specifications. Critically, no recompilation or code changes are required to incorporate new fields or form elements. Changes to presentation elements (widgets) or validation logic can all be made at runtime.

### Schema-less storage

A relational database is too restrictive to enable runtime modification of fields. Instead, a schema-less data store, MongoDB, was used to allow storage of dynamically defined data in the RDRF. MongoDB was chosen because it is an industry recognised schema-less data store with a powerful, well-documented query API. MongoDB stores all data in the form of “collections”, which are essentially lists of object dictionaries. Each DE captured by forms within a registry is stored as a single key value pair in one such dictionary. The key includes the patient identifier, the DE code and some additional bookkeeping to encode the registry form structure. To simplify the implementation of permissions, each user-defined registry is isolated in its own MongoDB data collection.

Metadata about each DE, including its data type, is stored as part of the DE definition in the relational SQL database. In this way, the relational SQL database and the NoSQL database provide a clean separation between static field-definition features (SQL) and the dynamic domain-level features (NoSQL).

### Creating multiple patient registries

**Figure 1:**
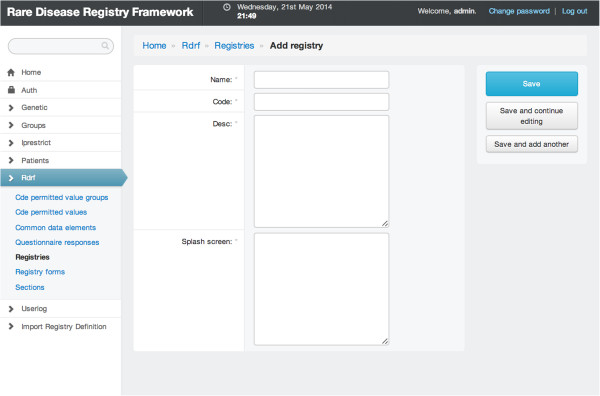
**Registry capture screen.** A screen capture of the form to create a new registry.

Figure 1 provides a screen capture of the registry creation screen. A user with appropriate credentials can use the web-based administration interface to create one or more registries.

### Data element (DE) definition and reuse

**Figure 2:**
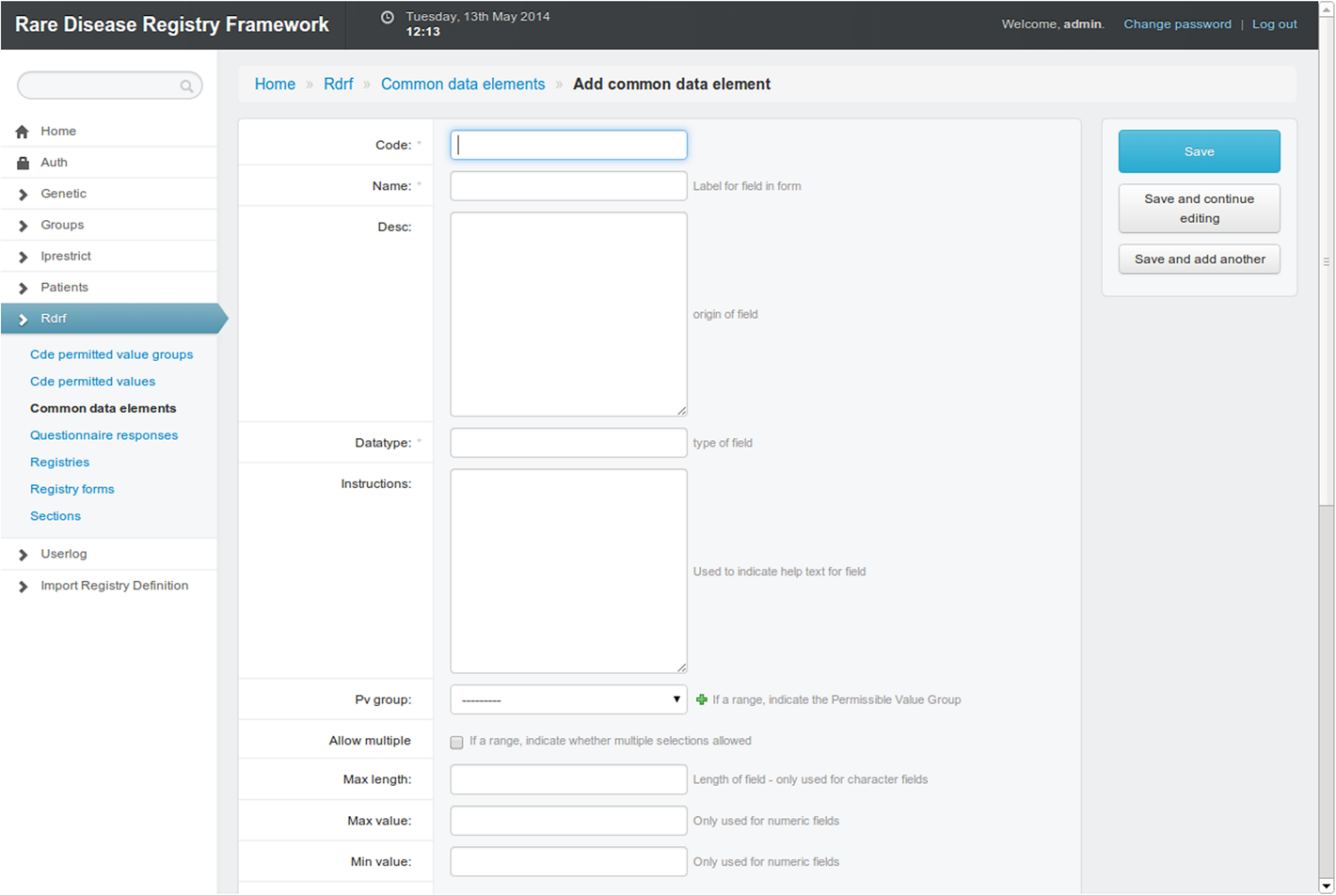
**Data elements definitions.** A screen capture of the form that appears to create a data elements.

Users can define data elements (DEs) that can be shared by all registries created within the framework. There are a number of attributes that can be set for DEs. There are multiple data types: integer, float, string, date, file, that can be used for attributes within each DE. Validation rules (e.g. minimum and maximum for numeric fields) can be defined and pattern validation can be defined for textual fields (e.g. credit-card number pattern consisting of four sets of four digits separated by a space). For more sophisticated DEs, a DE can incorporate Permitted Value Groups (PVG). For example, a PVG might be called, Size with the permissible values: large, medium, small. This PVG can then be applied to any DEs that requires the Size PVG. Figure 2 displays a screen capture of the DE definition page.

### Derived data elements (DDEs)

**Figure 3:**
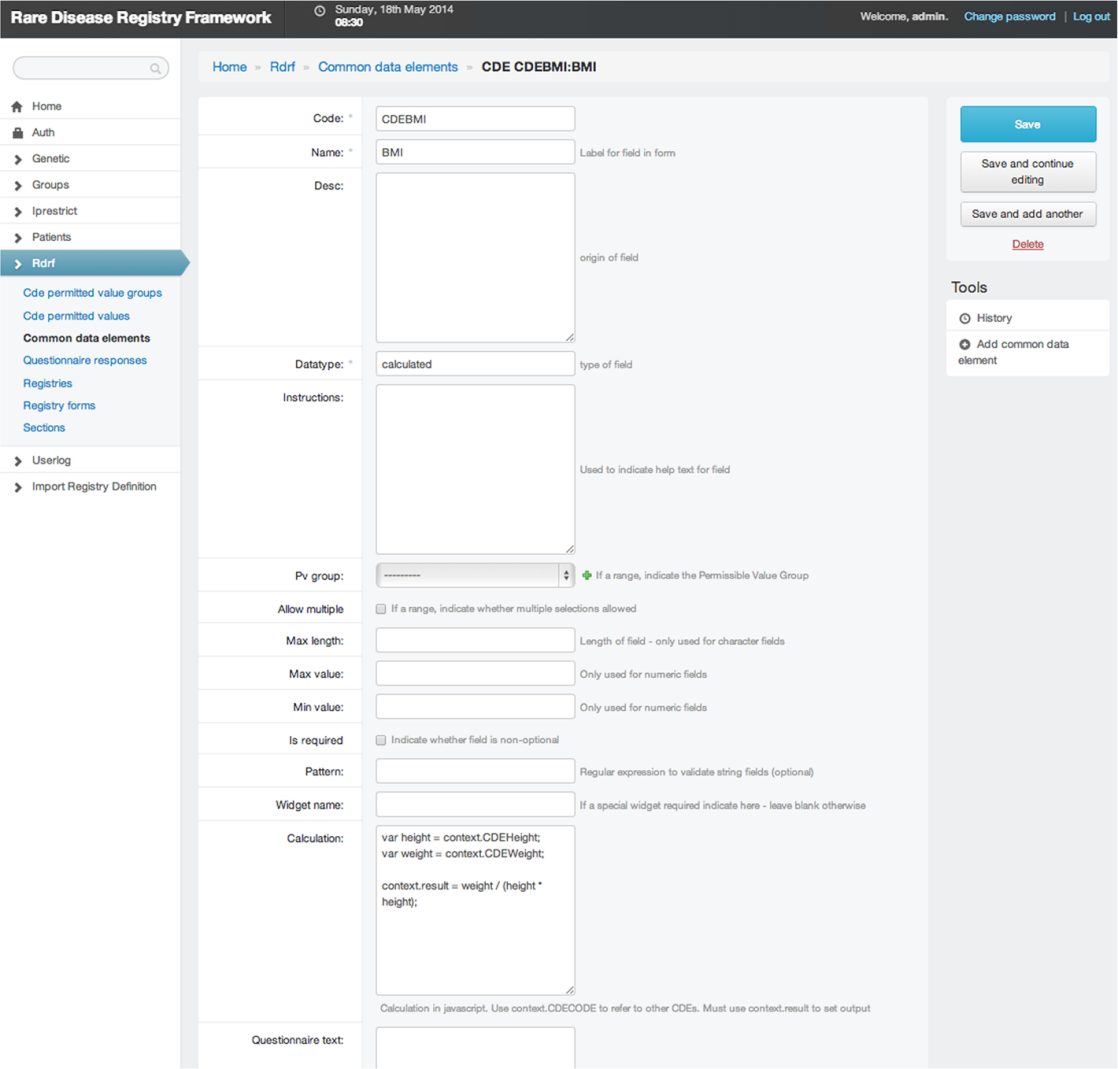
**Derived data elements.** A screen capture of a derived data element (Body Mass Index), derived from the two data elements: height and weight.

Within the second generation RDRF it is possible to create what we refer to as a derived data element (DDE). A typical example of a DDE is Body Mass Index (BMI) which is based on a calculation involving a Height DE and Weight DE. In the RDRF a DDE is defined using Javascript to represent the calculation involving one or more DEs. DDE are calculated dynamically and are therefore updated as soon as the DEs in the calculation are modified. Figure 3 is a screen capture of the DDE for BMI. In summary, the DE template can enable the creation of sophisticated DEs without the need for any software developer effort. DDEs can be similarly defined with the addition of a Javascript snippet to dynamically calculate the value of the DDE.

### Importing and exporting data elements

It is possible to import and export DEs via a YAML specification file. By using YAML, a single human-readable file definition of a registry can be exported which can then be version-controlled and used for sharing with other research communities.

A catalogue of all the DE defined within a patient registry is also exportable.

### File uploading

Registries often require documents to be uploaded into the system. Such forms include signed patient consent forms in a human health context or an export certification form in a biosecurity context. This can be simply achieved within the RDRF. Files can be uploaded and downloaded using the File fields after creating the specific file upload DE.

#### Demonstration registry

**Figure 4:**
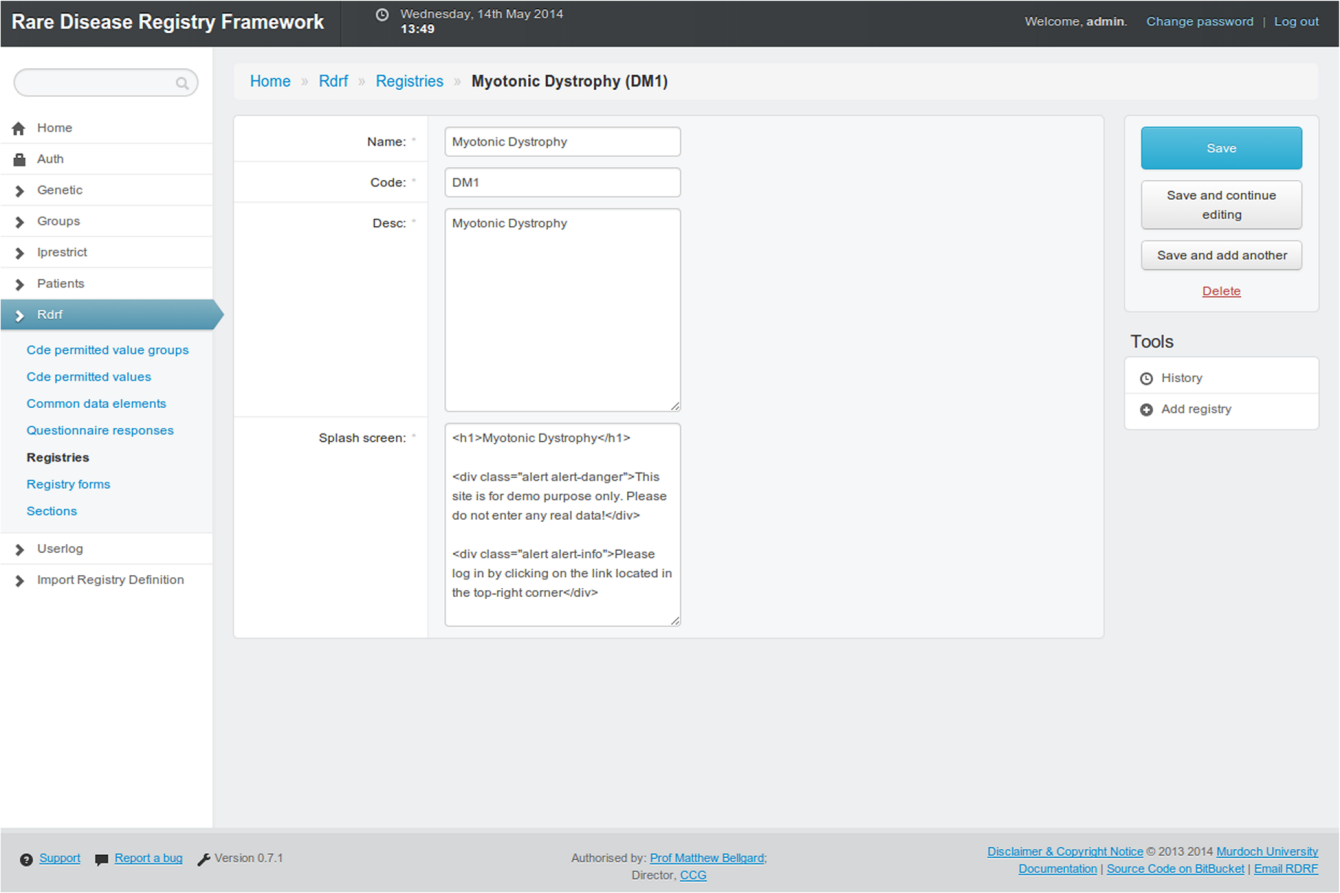
**Demonstration registry.** A screen capture of a patient questionnaire form for the Myotonic Dystrophy (DM1) demonstration registry (https://ccgapps.com.au/demo-rdrf/).

A demonstration rare disease registry using the second generation RDRF is available for testing (https://ccgapps.com.au/demo-rdrf/). The demonstration system allows the creation of new registries, modification of existing ones, creation of new data elements and so forth. The demonstration system has multiple accounts to allow users to test all levels of access and functionality (for example, login: admin, password: admin; login: curator, password: curator; login: genetic, password: genetic; and login: clinical, password: clinical). A screenshot of a patient questionnaire from the demonstration registry is shown in Figure 4.

## Discussion and conclusion

In this paper, we introduce significant enhancement to our patient registry framework (RDRF) which now allows the dynamic creation of new registries with little or no software development. This addresses the critical bottlenecks of community uptake and wider applicability. RDRF could also be used in a surveillance context or in clinical studies where there is an initial need to establish a registry with a given set of data elements (DEs) but will need to be later enriched with new DEs as a result of additional phenotypical measurements that need to be captured. Importantly, DEs are able to be created once and reused (shared) in multiple registries and can be one of multiple data types. From a systems perspective, we contend that shared DEs (SDEs) will enable higher levels of interoperability [4] that can facilitate ease of sharing and extensive data mining of patient data across jurisdictional boundaries - another significant bottleneck in patient disease registries. We introduce the concept of a derived DE (DDE). A DDE can be derived from one or more DEs which means it is possible to actualise efforts emanating from efforts to define a DE ontology (DEO). Current data standards for DEs (such as [8]) could be revised to reflect that the unique identifier used for any given DDEs, references the identifiers of DEs from which the DDE is derived.

From a technological perspective, the feature being exploited in the Python programming language is the ability to create classes dynamically. Typically most web applications define classes statically, whereas RDRF is dynamically constructing these classes at run time from the DE definitions, which act as the specification. We believe RDRF will have broader applicability and can now be used in a wide range of disease and surveillance contexts for human, animal, plant health or in a biosecurity context. RDRF will enable decision makers to establish registries thereby ensuring personal electronic records can be ‘born digital’. As RDRF now employs YAML, a single human-readable file definition of a registry can be exported. This means that it will be possible to version-control a registry for future sharing with other research communities or customising for specific needs.

In future releases we plan a number of enhancements, including: the definition of a DE specification format (DESF); ability to apply rules to the DEs to enable knowledge capture; and import outputs from a next generation sequencing experiment which includes both the variant call format (VCF) file as well as the analysis workflow audit trail [9].

## Availability and requirements

Project name: RDRF

Project home page: https://bitbucket.org/ccgmurdoch/rdrf

Documentation: http://rare-disease-registry-framework.readthedocs.org/

Operating system(s): Linux (CentOS 6 tested)

Programming language: Python

Other requirements: PostgreSQL, MongoDB, Apache (including mod_wsgi)

License: GNU GPL v3

Any restrictions to use by non-academics: Yes

## Abbreviations

DE: Data element; DEO: Data element ontology; DDE: Defined data element; DESF: Data element specification format; SDE: Shared data elements; RDRF: Rare disease registry framework.

## Competing interests

The authors declare that they have no competing interests.

## Authors’ contributions

MB and AH devised original concept design. Software architectural design LR, AH, MR. Wrote manuscript: MB, AH, LR. All authors read and approved the final manuscript.

## Authors’ information

Matthew Bellgard: Professor Matthew Bellgard (BSc Hons, PhD in Computer Science) is Murdoch University’s Bioinformatics Chair and the Director of the Western Australian State Government Centre of Excellence, the Centre for Comparative Genomics (CCG). His scientific work has resulted in developments in the areas of pairwise sequence alignment and artificial intelligence, early detection of base composition differences in closely related bacterial species, whole genome sequence analysis and advances in the development of web-based integrated systems utilising high performance computing.

Lee Render: BSc Physics UWA, BA Philosophy (Hons) UWA. Has over ten years industry experience in software development with experience in .NET and Java. He has had direct experience in designing, developing and maintaining bioinformatics workflow systems. Current focus on Python software development.

Maciej Radochonski: Maciej Radochonski completed a MSc Eng in Applied Computer Engineering at Wroclaw University of Technology, Poland and a MSc in Information and Communication Technology at The University of Notre Dame Australia. Maciej has nearly 8 years experience in software development using Java and related technologies. Current focus on Python software development.

Adam Hunter: Adam Hunter completed a BSc Honours in Computer Science at Murdoch University. Adam has over 10 years experience in ICT including software development in C and Java. He leads the CCG software development and infrastructure team. Current areas of focus include continuous integration, agile programming and cloud computing.
